# Pedicled flap transfer after chest wall malignant tumor resection and potential risk of postoperative respiratory problems for patients with low FEV1.0%

**DOI:** 10.3389/fsurg.2024.1357265

**Published:** 2024-03-05

**Authors:** Kunihiro Asanuma, Masaya Tsujii, Tomohito Hagi, Tomoki Nakamura, Teruya Uchiyama, Ryohei Adachi, Kenta Nakata, Takeshi Kataoka, Akihiro Sudo

**Affiliations:** Department of Orthopedic Surgery, Mie University School of Medicine, Tsu City, Japan

**Keywords:** chest wall, malignant tumor, pedicle flap, respiratory complication, FEV1.0%

## Abstract

**Introduction:**

Musculoskeletal transfer for chest wall tissue defects is a crucial method, and pedicled flaps around the chest wall are preferred in terms of location and simplicity of transfer. These require special care because of complications such as partial necrosis, fistula, wound dehiscence, infection, hematoma and restricted function of the arm or shoulder. However, studies of respiratory function are rare. In the present study, we investigated the complications including respiratory problems after wide resection for malignant chest wall tumors with musculoskeletal pedicle transfer.

**Methods:**

A total of 13 patients (15 operations) who underwent wide resection of primary, recurrent, or metastatic malignant chest wall tumors and musculoskeletal pedicle transfer for coverage of tissue defects were enrolled in the present study. A retrospective review of all patients was performed using data collected from hospital records and follow-up information. The complications of musculoskeletal transfer after chest wall wide resection, including respiratory problems, are evaluated.

**Results:**

Rib or sternal resection was performed in 12 operations, and only soft tissue resection was performed in 3 operations. Latissimus dorsi (LD) pedicle transfer was performed in 13 operations, and pectoralis major (PM) pedicle transfer was performed in 2 operations; basically, wounds were closed primarily. Surgical complications were observed following 5 of the 15 operations (33.3%). Respiratory complications were seen in 7 of the 15 operations (46.7%). Patients with respiratory complications showed significantly lower preoperative FEV1.0% values than those without respiratory complications (*p* = 0.0196). Skin resection area tended to be higher in the complication group than in the no complication group (*p* = 0.104).

**Discussion:**

Pedicled myocutaneous flap transfers such as LD, PM, and rectus abdominus can be used following multiple resections. After harvesting LD or PM, the wound can be closed primarily for an 8–10-cm skin defect in patients with normal respiratory function. However, for patients with low FEV1.0%, after primary closure of LD or PM transfer for wide soft tissue defects, attention should be paid to postoperative respiratory complications.

## 1 Introduction

Malignant tumors of the chest wall account for approximately 5% of all thoracic malignancies (1). Management of these tumors often involves a multidisciplinary approach encompassing wide local excision for complete removal of the tumor, which often necessitates chest wall reconstruction to preserve structural integrity and respiratory function and protect the underlying organs. The chest wall consists of many layers of various tissues, including skin, fat, muscles, rib bones, and pleura. Resected tissues vary based on the location and depth of tumors; therefore, the reconstruction strategy for chest wall and soft tissue coverage is a considerable problem. For superficial tumors, soft tissue transfer is often needed for a wide soft tissue defect. For deep tumors, a wide rib or sternal defect needs non-rigid or rigid reconstruction by various artificial materials, and sufficient soft tissue coverage is occasionally required. Commonly, prosthetic materials are provided after resection of three or more ribs, and reconstruction of posterior defects is not necessary except with defects over 10 cm and scapular trapping (2). We previously suggested that posterior bony defects lead to scoliosis even after one rib resection, and rigid reconstruction may help to relax scoliosis (3). In general, the reconstruction strategy selected depends on the experience and technique of the surgeons (4, 5).

Musculoskeletal transfer for chest wall tissue defects is a crucial method to restore stability, protect the underlying organs or defend materials from bacterial infections, and promote a better quality of life for the patient. Several types of flaps are commonly used for musculoskeletal transfer in chest wall reconstruction, including the latissimus dorsi flap (LD), pectoralis major flap (PM), rectus abdominis (RA) flap, omental flap, external oblique flap, serratus anterior flap, fascia lata free flap, and anterolateral thigh free flap (6–8). In these cases, pedicled flaps around the chest wall are preferred in terms of location and simplicity of transfer. In particular, LD flaps and PM flaps are among the most commonly used flaps in chest wall reconstruction (8, 9). These require special care because of complications such as partial necrosis, fistula, wound dehiscence, infection, and hematoma. In addition, though restricted function of the arm or shoulder has been reported (10), studies of respiratory function are rare.

In the present study, we investigated the complications including respiratory problems after wide resection for primary, recurrent, or metastatic malignant chest wall tumors with musculoskeletal pedicle transfer of LD or PM.

## 2 Methods

A total of 13 patients (15 operations) who underwent wide resection of primary, recurrent, or metastatic malignant chest wall tumors and musculoskeletal pedicle transfer for coverage of tissue defects between 1997 and 2021 at Mie University Hospital were enrolled in the present study. Histopathological diagnoses were verified by independent pathologists.

A retrospective review of all patients was performed using data collected from hospital records and follow-up information. The present study was approved by the Ethics Committee of the Mie University Graduate School of Medicine (approval number: H2020-224). All procedures performed in studies involving human participants were undertaken in accordance with the ethical standards of the Ethics Committee of Mie University and with the 1975 Declaration of Helsinki.

In three operations in which only soft tissues were resected, only flap transfers were performed. Resections involving 2 or more ribs were reconstructed with BARD mesh (Bard, Cranston, RI) or BARD Composix mesh (Bard) with flap transfer for eight operations. Excess mesh was trimmed, and 1-0 nylon sutures were tightened between bone or soft tissue as much as possible and covered by pedicle flap. For the four operations of sternal resection, over 5 cm in size by rib resections or re-resection of ribs due to recurrence, rigid reconstruction was performed (Table 3). The modified method is applied to its reconstruction as follows. Polymethylmethacrylate (PMMA, Simplex P; Stryker Howmedica Osteonics, Mahwah, NJ) remodeled smaller than the chest wall bone defect was sandwiched by a double-layer or quadri-layer BARD mesh. The mesh around the PMMA was sutured to fix the prosthetic material in place using 3-0 prolene or nylon. Excess mesh was trimmed, and 1-0 nylon sutures were tightened between the bone or soft tissue and mesh. Flap reconstruction was performed by LD or PM.

Preoperative respiratory function was tested before 15 operations. Respiratory functions were evaluated using percentage predicted forced expiratory volume in 1 s (FEV1.0%, FEV1/FVC) and percent vital capacity (%VC, VC/predicted VC).

### 2.1 Statistical analysis

Statistical evaluations were performed to compare various parameters, using the Mann-Whitney test for quantitative data analysis, with the level of significance set to *p*-values less than 0.05. All statistical evaluations were performed with the aid of the EZR software application (11).

## 3 Results

### 3.1 Patient and tumor characteristics

A total of 13 patients (10 males, 3 females) and 15 operations, including 8 operations for primary tumors, 4 operations for recurrent tumors, 2 operations for metastatic tumors, and 1 operation for tumors that had been resected inadequately in a previous hospital, were analyzed. The histopathological diagnoses were myxofibrosarcoma (3), leiomyosarcoma (2), chondrosarcoma (2), liposarcoma (2: dedifferentiated 1, pleomorphic 1), synovial sarcoma (1), extraskeletal osteosarcoma (1), and meningioma (1) (Table 1). The mean age was 66.3 years (range, 41–84 years), and mean tumor size was 9.4 (range, 5–23) cm (Table 2). All patients underwent wide resection and pedicled flap transfer. One patient underwent 3 resections for recurrent tumor and 3 pedicled flap transfers. Tumor location was anterior in 10 tumors, lateral in 4 tumors, and posterior in 1 tumor. The mean duration of follow-up was 32.6 (range, 5–146) months.

**Table 1:** Histopathological diagnoses.

Histology	*N*=
Myxofibrosarcoma	3
Leiomyosarcoma	2
Chondrosarcoma	2
MPNST	1
Dedifferntiated liposarcoma	1
Pleomorphic liposarcoma	1
Synovial sarcoma	1
Extraskeletal osteosarcoma	1
Meningioma	1

**Table 2:** Operative information 1.

Patient no.	Age	Gender	Tumor size		Histology	Tumor status	Location
1	68	Male	7	Soft tissue	Liosarcoma	Primary	Ante
2	84	Male	5.6	Soft tissue	Myxofibrosarcoma	Primary	Ante
3	61	Male	8	Soft tissue	Liposarcoma	Recurrence	Ante
4	52	Male	5.7	Bone	Chondrosarcoma	Primary	Ante
5 Ope-1	70	Male	6	Soft tissue	MPNST	Recurrence	Late
Ope-2	71		6.6			Recurrence	Ante
Ope-3	74		6			Recurrence	Ante
6	71	Female	9	Soft tissue	Myxofibrosarcoma	Primary	Late
7	50	Male	23	Bone	Chondrosarcoma	Primary	Late
8	66	Male	10	Soft tissue	Leiomyosarcoma	Primary	Post
9	78	Male	11.5	Soft tissue	Extraskeletal osteosarcoma	Primary	Late
10	71	Male	5	Soft tissue	Leiomyosarcoma	Other hosp	Ante
11	41	Female	5.6	Soft tissue	Synovial sarcoma	Meta	Ante
12	70	Male	20	Soft tissue	Myxofibrosarcoma	Primary	Ante
13	68	Female	12.5	Bone	Meningioma	Meta	Ante

### 3.2 Operative information

Rib resection was performed in 8 operations, and the mean number of resected ribs was 4.2 (range, 2–7). The sternum was resected in 4 operations. Only superficial tissue resection was performed in 3 operations. In bone resection procedures, reconstruction was performed by mesh in 8 operations and by mesh and PMMA in 4 operations. The mean skin resection area was 101 (range, 0–408) cm^2^, and the mean chest wall resection area was 102 (range, 0–387) cm^2^. LD pedicle transfer was performed in 13 operations, and PM pedicle transfer was performed in 2 operations. One patient underwent full thickness chest wall resection 3 times due to recurrence of a malignant peripheral nerve sheath tumor (MPNST) (patient No. 5). In the present study, donor and recipient sites were basically closed primarily without skin graft, except in one operation. Mean bleeding was 453 (range, 50–3,170) ml. Mean operation time was 7.7 (range, 4–12.5) h. Radiation therapy was administered in relation to 3 operations (Table 3).

**Table 3:** Operative information 2.

Patient no.	Bone resection	Skin resection area (cm^2^)	Chest wall resection area (cm^2^)	Flap	Prosthesis	Bleeding (ml)	Operation time (h)	Radiation
1	—	84.8	0	LD	—	57	4.7	
2	—	106.8	0	LD	—	208	4.3	35Gy/5fr
3	—	112.3	0	LD + skin graft	—	50	5.3	50Gy/25
4	2 ribs	5.7	45.7	PM	Mesh + PMMA	111	5.4	
5 Ope-1	4 ribs	106	115.04	LD	Mesh	130	7.2	
Ope-2	3 ribs	142.9	112.12	PM	Mesh	52	7.2	
Ope-3	3 ribs	146.8	109.41	LD	Mesh + PMMA	720	9.7	70Gy/35fr
6	4 ribs	0	127.96	LD	Mesh	94	4.0	
7	4 ribs	408.2	134.03	LD	Mesh	700	11.0	
8	7 ribs	0	75.74	LD	Mesh	3,170	12.5	
9	7 ribs	86.4	386.99	LD	Mesh + PMMA	568	9.6	
10	Sternum	113	119.59	LD	Mesh	206	8.7	
11	Sternum	25.1	100.61	LD	Mesh + PMMA	400	9.4	
12	Sternum	153.1	131.45	LD	Mesh	231	9.4	
13	Sternum	25.1	77.11	LD	Mesh	100	7.6	

### 3.3 Respiratory function

**Figure 1:**
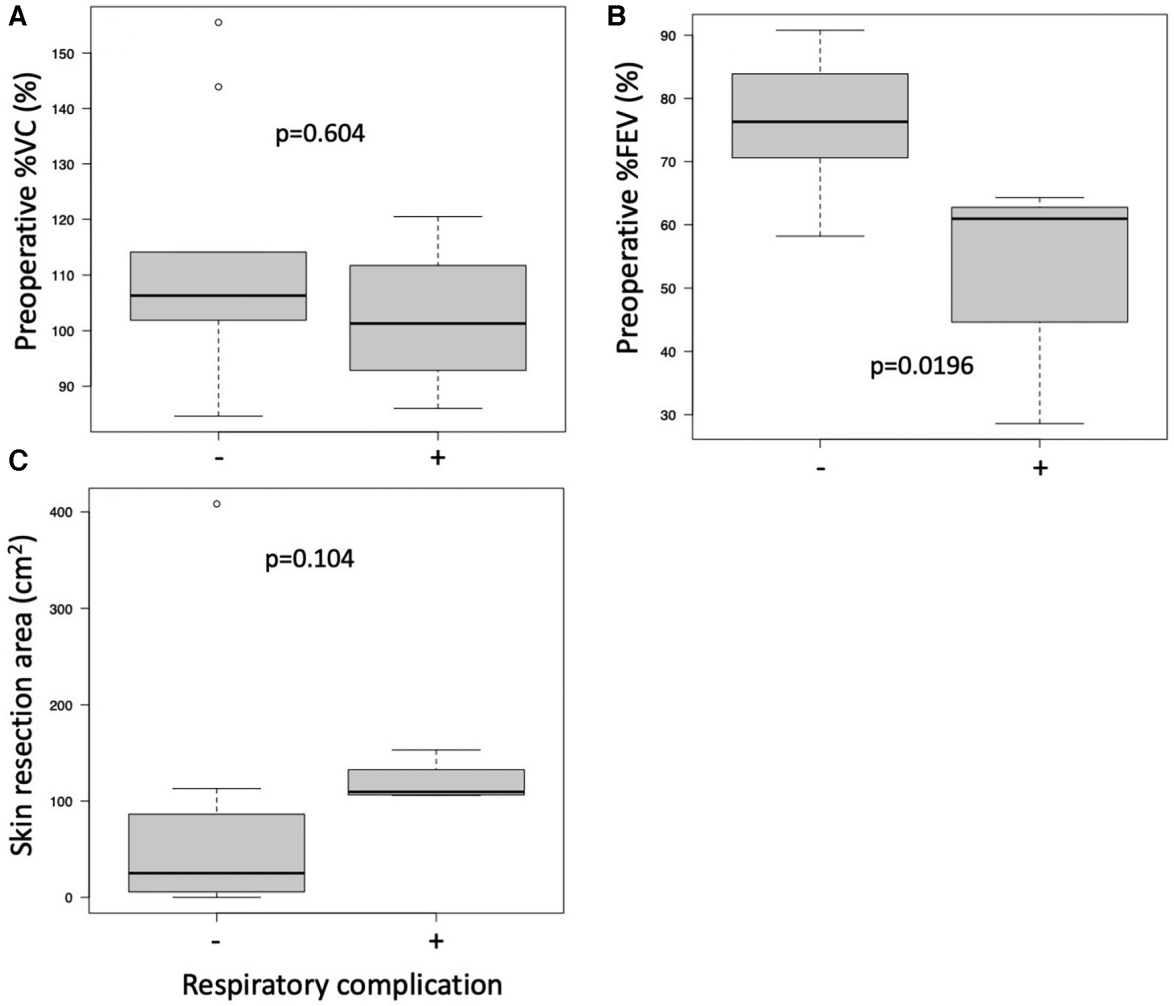
Operation data. %VC (**A**), FEV1.0% (**B**) and skin resection area (**C**) are compared between patients with and without respiratory complications.

Respiratory function was evaluated by preoperative %VC and %FEV. Mean preoperative %VC and FEV1.0% were 104.9% (range, 74.7–155.5%) and 67% (range, 28.6–90.8%), respectively. Respiratory complications were seen in 7 of the 15 operations (46.7%), with postoperative shortness of breath in 6 cases. Postoperative noninvasive positive-pressure ventilation for 12 h was performed in 1 case (Table 4). Preoperative %VC was not different between the patients with and without respiratory complications (Figure 1A, *p* = 0.604). Patients with respiratory complications had significantly lower preoperative FEV1.0% than those without respiratory complications (Figure 1B, *p* = 0.0196). Skin resection area tended to be greater in the complication group than in the no complication group (Figure 1C, *p* = 0.104).

**Table 4:** Operative information 3.

Patient no.	Surgical complication	Preoperative %VC	Preoperation %FEV1.0	Respiratory complication
1		101.9	68.5	
2		120.5	60.7	Shortness of breath
3		86	28.6	Shortness of breath
4		107.7	83.9	
5 Ope-1		102.9	61.2	Shortness of breath
Ope-2		86.5	48.6	Shortness of breath
Ope-3		74.7	52.1	Shortness of breath
6	Hematoma Chronic seroma	106.3	82	
7	Small necrosis in flap	84.8	90.8	
8	Infection	143.9	72.5	
9			84.6	76.3
10		155.5	70.6	
11	Cardiac effusion	104	86.4	
12		99.7	64.3	Shortness of breath
13	Noninvasive positive-pressure ventilation	114.1	58.2	Noninvasive positive-pressure ventilation

### 3.4 Surgical complications

Surgical complications were observed following 5 of the 15 operations (33.3%). Infection occurred in 1 case that was reconstructed by BARD mesh and LD after 7-rib resection. The infection resolved within 2 months by washing, antibiotics, and negative pressure wound therapy. A pericardial effusion was seen in 1 case reconstructed by BARD mesh, PMMA, and LD after sternal resection. The patient recovered with a thoracic drainage tube for 5 days. Respiratory failure was seen in 1 case that was reconstructed by BARD mesh and LD after sternal resection. After extubation, noninvasive positive-pressure ventilation was initiated for only 12 h. Small necrosis in the flap was seen in 1 case reconstructed by BARD mesh and LD. Debridement and wound closure were performed 6 days after resection. Hematoma and chronic seroma were seen in 1 case reconstructed by mesh and LD after 4-rib resection. The patient recovered with thoracic drainage for 5 days (Table 4).

## 4 Discussion

Myocutaneous flap transfer remains one of the gold standards for chest wall reconstruction after resection. The aim of a flap after chest wall resection is to fill tissue defects, cover the prosthesis, protect organs, form the thoracic cavity, and close operative wounds. The choice of flap is dependent on the size of the tissue defect, defect location, donor availability, and previous operations, and operative time and surgical complexity affect surgical outcomes. Pedicled flaps involve a simpler surgical technique because they maintain their original blood supply. In contrast, free flap transfer is a more technically challenging and time-consuming procedure, requiring microvascular anastomosis to ensure flap survival. In a retrospective analysis of 628 head and neck cancer patients, the major flap complication rate was 5.6% with free flaps and 2.1% with pedicled flaps (12). A study of 171 oral cavity cancer patients showed that, whereas the donor site complication rate was significantly higher with a free flap (34.2%) than with a pedicled flap (11.2%), the rate at the recipient site was no different (13). Another study of 133 flap reconstructions of full-thickness chest wall defects showed that postoperative complications were not significantly different between free (*n* = 28) and pedicled flaps (*n* = 105) (14). Pedicled flaps generally offer specific advantages, such as reliability, technical simplicity, shorter operative time, and reduced risk of vascular compromise, making them a valuable tool in chest wall reconstruction post-resection compared with free flaps.

LD, PM, and RA are the most commonly used pedicled flaps in reconstruction of chest wall defects (7–9). The LD muscle and musculocutaneous flaps are ideally suited for reconstruction of large anterior, anterolateral, and posterior thoracic defects. With an LD flap, up to 35 cm^2^ × 20 cm^2^ of muscle flap can be harvested (8). The reported size of the skin paddle was up to 28 cm^2^ × 17 cm^2^ (15). Wounds of the donor site up to 8–10 cm in width can be closed primarily (10, 16). PM was commonly used as a muscle advancement or rotation flap for chest wall reconstruction. A PM flap can cover anterior, axillary, lateral chest wall, and supraclavicular defects (8). The reported large size of the skin paddle of the PM flap was 15 cm^2^ × 10 cm^2^ (17). The RA flap is pedicled by the superior epigastric artery and used for anterior to anterior-lateral chest wall defects. With a skin paddle, the vertical rectus abdominis myocutaneous (VRAM) flap, transverse rectus abdominis myocutaneous (TRAM) flap or T-inverted shaped rectus abdominis myocutaneous (Ti-RAM) flap can be appropriate (6, 8, 18–20).

**Figure 2:**
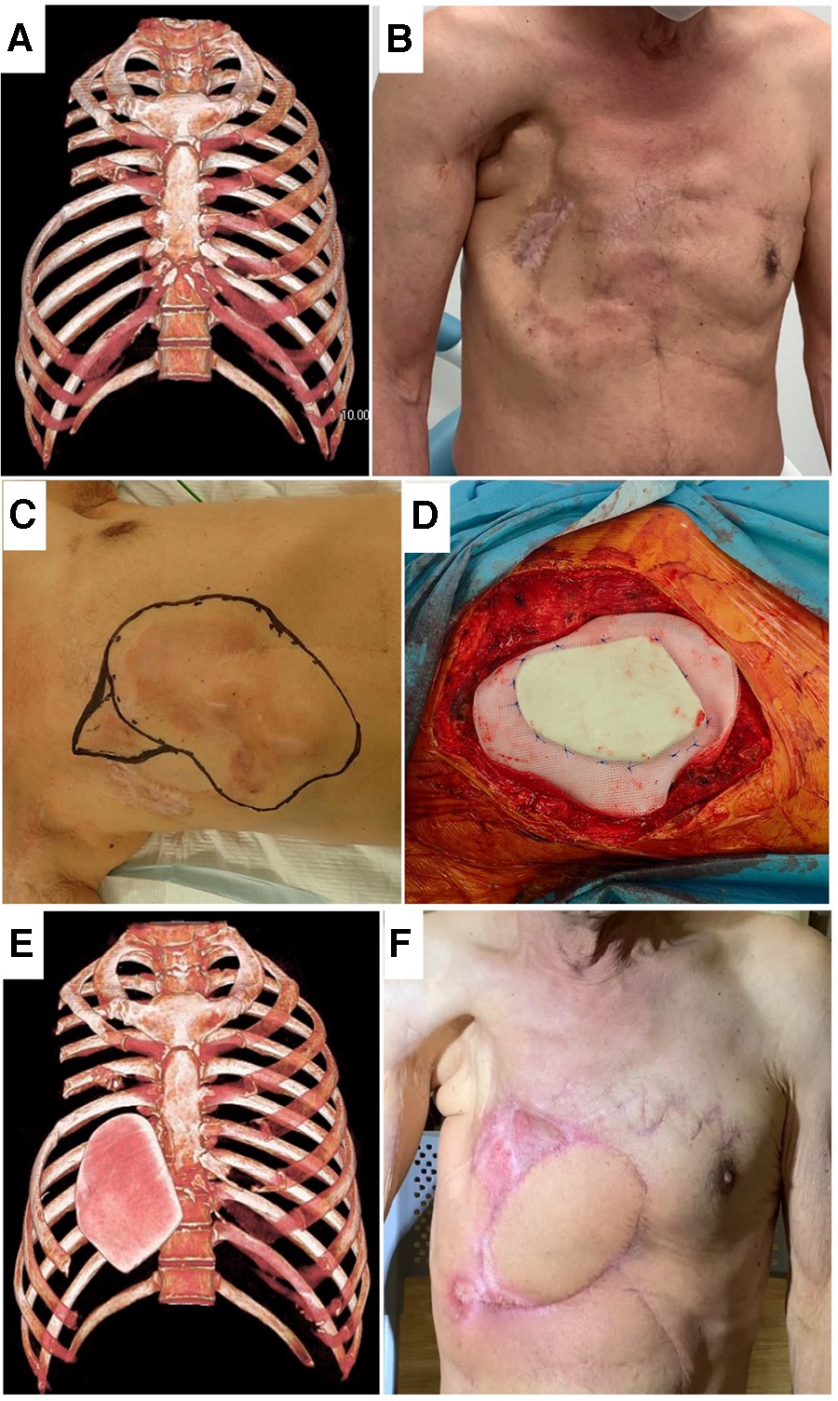
Patient No. 5. Frontal view of 3D-CT (**A**) and postoperative picture (**B**) after two chest wall resections. Resection area for recurrence (**C**), covered by PMMA and polypropylene complex (**D**), frontal view of 3D-CT with PMMA (**E**) and postoperative picture (**F**) in the third resection.

In the present study, LD was mainly used, for 13 operations, and PM was used for 2 operations. In patient 5, full thickness resection was performed 3 times, and bilateral LD (Table 3. Patient No. 5-1, 5-3) and the left PM (Table 3. Patient 5-2) were used for each reconstruction. In this patient, repeated chest wall resections aggravated the flail chest (Figures 2A,B, Video left). Some authors said that nonrigid reconstruction was sufficient instead of rigid reconstruction (21). However, rigid reconstruction by the sandwich method improved the flail chest (Figures 2C–F, Video, right), which was stable for 3 years. We previously reported the higher survival rate of the sandwich method than the titanium plate, and the sandwich method was our preferred approach (3). In the present case, the superior epigastric artery was ligated at liver resection. If the artery is intact, an RA flap can be used. Pedicled flaps can cover multiple chest wall resections.

**Figure 3:**
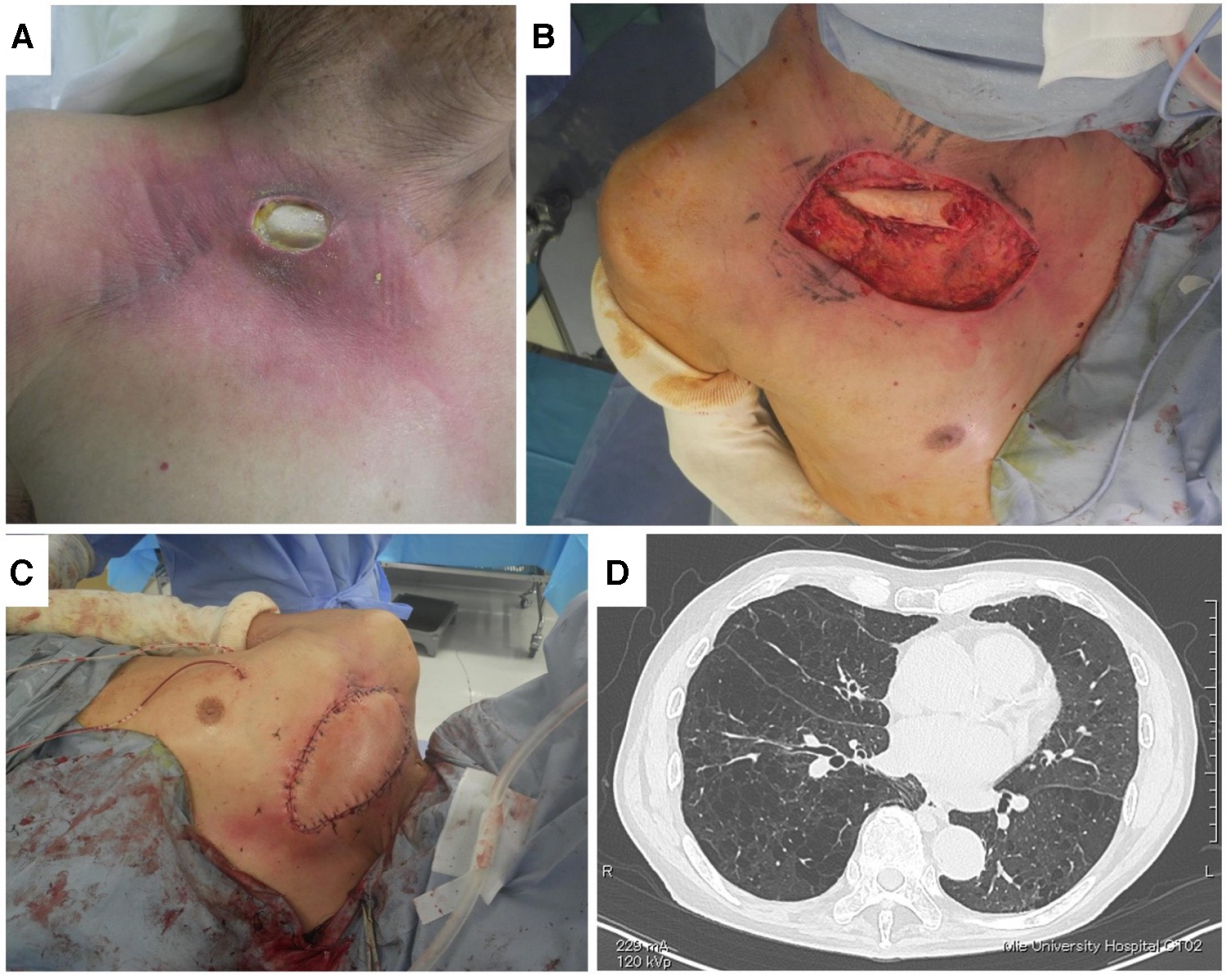
Patient No. 2. Fistula (**A**), debridement of infected and irradiated area (**B**), coverage by right LD (**C**), and lung CT (**D**).

Patients with pre-existing pulmonary conditions might be at increased risk of postoperative pulmonary complications, including pneumonia and respiratory failure; bony resection and reconstruction following chest wall tumor resection affect pulmonary conditions directly (3, 22, 23). In the present series, respiratory problems occurred in 7 of 15 operations. Notably, two of seven who had respiratory problems were superficial, and only soft tissues were resected. In patient 2, LD was transferred without bony chest wall resection, and the donor site was closed primarily. Postoperatively, the patient complained of shortness of breath and limitation of chest expansion (Figures 3A–D). Given this, we hypothesized that primary closure in the donor and recipient site using LD or PM results in lack of soft tissues around the chest and may lead to limitations of chest expansion at inspiration.

**Figure 4:**
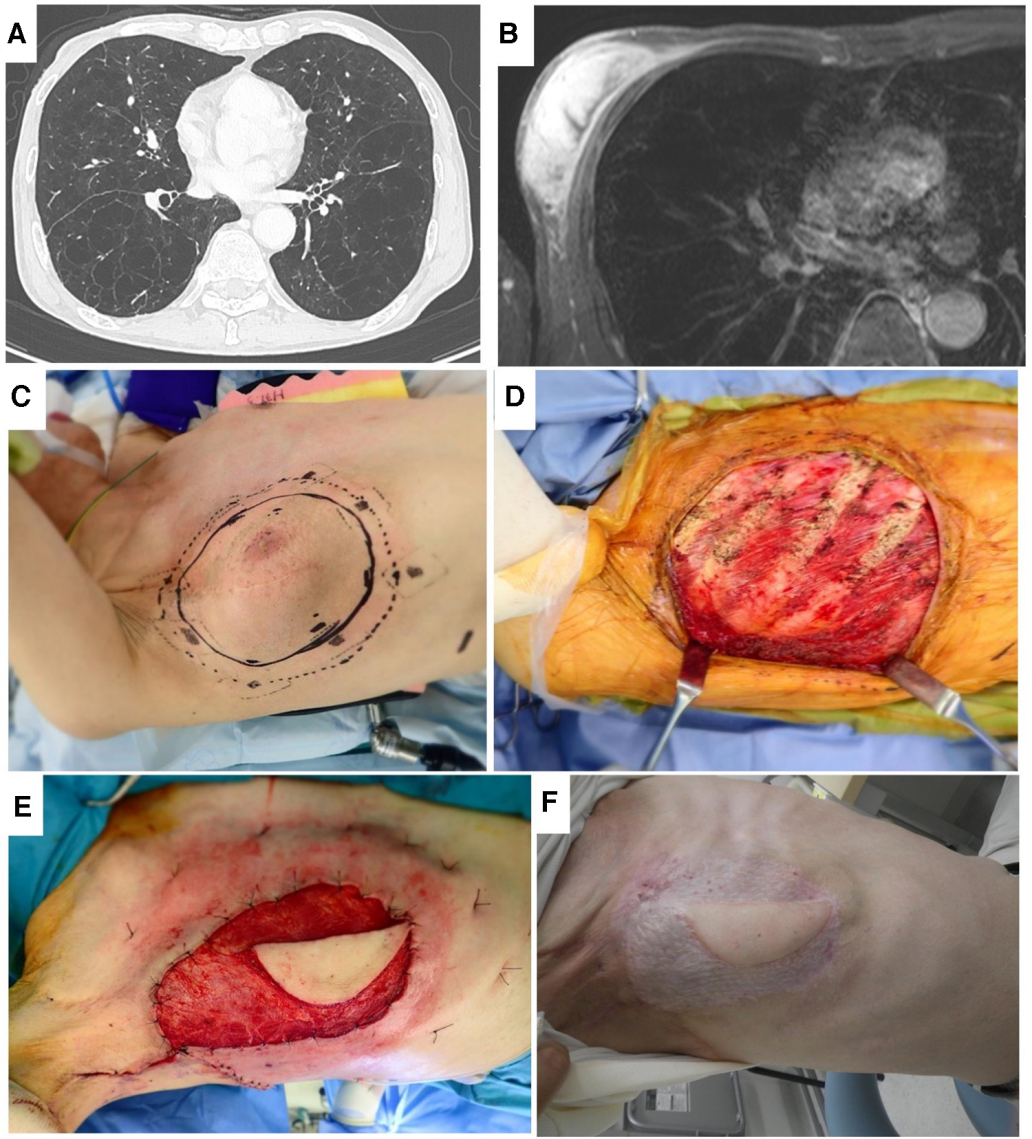
Patient No. 3. Lung CT (**A**), axial MRI (**B**), resection area (**C**), after resection (**D**), coverage by same-side LD before skin graft (**E**), and healed by skin graft (**F**).

In their series of PM transfer for head and neck cancer, Talmi et al. reported that PM harvesting and donor site closure may be related to decreased postoperative FVC. They also suggested that methods of donor site closure should be considered for patients with preoperative pulmonary problems (24). In the present data, in the respiratory complication group, FEV1.0% was significantly lower than in the no complication group (Figure 1B, *p* = 0.0196). We considered that primary closure after harvesting LD or PM for patients with low FEV1.0% probably resulted in symptoms. We changed our ordinary method in patient 3 due to the severely low FEV1.0% (28.6%) (Figure 4A). Although at least 3-rib resection was needed for complete resection (Figure 4B), full thickness resection was not permitted by the respirologist. Therefore, preoperative radiation therapy was performed, and the tumor was resected by marginal resection without rib resection (Figures 4C,D). For wide skin and muscle defects, the right LD was transferred with a skin graft (Figures 4E,F). The patient complained of shortness of breath for 3 months, but he recovered and can play golf without respiratory support.

In the present series, FEV1.0% was under 64.3% in all patients with respiratory complications. Patients with preoperative FEV1.0% <80% showed significant decreases in the Physical Component Summary score, and this was related to quality of life (25). From our experience, primary closure for soft tissue defects after flap transfer led to some limitation of chest expansion, and many cases were asymptomatic from the respiratory perspective. However, primary closure in patients with low FEV1.0% led to symptoms. Now, our criterion was to avoid primary closure for patients with low FEV1.0%. However, the threshold for low or high FEV1.0% could not be determined from the present data, but candidate thresholds for dividing FEV1.0% have been suggested, including 80% from Tacconi's study (25) and 70% related to COPD diagnosis (26). This needs further study. To avoid respiratory complications following myocutaneous flap transfers in patients with low FEV1.0%, several approaches can be considered: a skin graft to avoid tight wound closure when harvesting LD or PM flaps; abdominal flaps, because in the study of deep inferior epigastric perforator flaps for breast reconstruction, respiratory function was not negatively affected (27); free flap from fascia lata, anterolateral thigh, or similar, except for the chest wall; and preoperative inspiratory muscle training (28).

In conclusion, pedicled myocutaneous flap transfers provide a useful and valuable tool for chest wall reconstruction after resection. Bilateral LD, PM, and rectus abdominus flaps can be used for multiple resections. After harvesting LD or PM, the wound can be closed primarily for 8–10-cm skin defects for patients with normal respiratory function. However, for patients with low FEV1.0%, primary closure after LD or PM transfer for wide soft tissue defects has a risk of postoperative respiratory complaints and should be avoided. However, for patients with low FEV1.0%, after primary closure of LD or PM transfer for wide soft tissue defects, attention should be paid to postoperative respiratory complications.

## 5 Limitation

This study was a retrospective series of cases from a single institution. These operations were rare and the number of patients was small, and there are many pathological subtypes. We could not performed statistical analysis for each pathological subtype. However, we reported our experience with potential risk of postoperative respiratory problems by pedicled flap transfer for chest wall malignant tumor resection. The surgeons often tend to underestimate the risks associated with respiration by flap transfer. We believe that this study provides a cautionary note regarding the respiratory risks associated with chest wall flap surgery.

## Data Availability

The raw data supporting the conclusions of this article will be made available by the authors, without undue reservation.

## References

[1] Foroulis CN Kleontas AD Tagarakis G Nana C Alexiou I Grosomanidis V Massive chest wall resection and reconstruction for malignant disease. Onco Targets Ther. (2016) 9:2349. 10.2147/OTT.S101615 27143930 PMC4846065

[2] Wang L Yan X Zhao J Chen C Chen C Chen J Expert consensus on resection of chest wall tumors and chest wall reconstruction. Transl Lung Cancer Res. (2021) 10(11):4057–83. 10.21037/tlcr-21-935 35004239 PMC8674598

[3] Asanuma K Tsujii M Hagi T Nakamura T Kita K Shimamoto A Full-thickness chest wall resection for malignant chest wall tumors and postoperative problems. Front Oncol. (2023) 13:1104536. 10.3389/fonc.2023.1104536 37152065 PMC10160664

[4] Colella S Brandimarte A Marra R Marinari S D'Incecco A Di Genesio Pagliuca M Chest wall reconstruction in benign and malignant tumors with non-rigid materials: an overview. Front Surg. (2022) 9:976463. 10.3389/fsurg.2022.976463 35990091 PMC9381953

[5] Divisi D Tosi D Zaccagna G De Vico A Diotti C Crisci R. Case report: a new tool for anterior chest wall reconstruction after sternal resection for primary or secondary tumors. Front Surg. (2021) 8:691945. 10.3389/fsurg.2021.691945 34355015 PMC8331331

[6] Salo J Tukiainen E. Flap reconstruction of the chest wall after oncologic resection. Curr Chall Thoracic Surg. (2020) 2:5. 10.21037/ccts.2019.12.05

[7] Arnold PG Pairolero PC. Chest-wall reconstruction: an account of 500 consecutive patients. Plast Reconstr Surg. (1996) 98(5):804–10. 10.1097/00006534-199610000-00008 8823018

[8] Bakri K Mardini S Evans KK Carlsen BT Arnold PG. Workhorse flaps in chest wall reconstruction: the Pectoralis Major, Latissimus dorsi, and Rectus abdominis flaps. Semin Plast Surg. (2011) 25(1):43–54. 10.1055/s-0031-1275170 22294942 PMC3140231

[9] Hameed A Akhtar S Naqvi A Pervaiz Z. Reconstruction of complex chest wall defects by using polypropylene mesh and a pedicled Latissimus dorsi flap: a 6-year experience. J Plast Reconstr Aesthet Surg. (2008) 61(6):628–35. 10.1016/j.bjps.2007.04.011 17656168

[10] Zhang YX Messmer C Pang FK Ong YS Feng SQ Qian Y A novel design of the multilobed Latissimus dorsi myocutaneous flap to achieve primary donor-site closure in the reconstruction of large defects. Plast Reconstr Surg. (2013) 131(5):752e–8e. 10.1097/PRS.0b013e3182865bcc 23629114

[11] Kanda Y. Investigation of the freely available easy-to-use software ‘ezr’ for medical statistics. Bone Marrow Transplant. (2013) 48(3):452–8. 10.1038/bmt.2012.244 23208313 PMC3590441

[12] Katna R Girkar F Tarafdar D Bhosale B Singh S Agarwal S Pedicled flap vs. free flap reconstruction in head and neck cancers: clinical outcome analysis from a single surgical team. Indian J Surg Oncol. (2021) 12(3):472–6. 10.1007/s13193-021-01353-1 34658573 PMC8490574

[13] Sittitrai P Ruenmarkkaew D Klibngern H. Pedicled flaps versus free flaps for oral cavity cancer reconstruction: a comparison of complications, hospital costs, and functional outcomes. Int Arch Otorhinolaryngol. (2023) 27(1):e32–42. 10.1055/s-0042-1751001 36714904 PMC9879635

[14] Vanstraelen S Ali B Bains MS Shahzad F Allen RJ Jr Matros E The contribution of microvascular free flaps and pedicled flaps to successful chest wall surgery. J Thorac Cardiovasc Surg. (2023) 166(4):1262–72.e2. 10.1016/j.jtcvs.2023.05.018 37236598 PMC10528168

[15] Bailey SH Saint-Cyr M Oni G Wong C Maia M Nguyen V The low transverse extended Latissimus dorsi flap based on fat compartments of the back for breast reconstruction: anatomical study and clinical results. Plast Reconstr Surg. (2011) 128(5):382e–94e. 10.1097/PRS.0b013e31822b7a3b 22030499

[16] Yuen AP Ng RW. Surgical techniques and results of lateral thoracic cutaneous, myocutaneous, and conjoint flaps for head and neck reconstruction. Laryngoscope. (2007) 117(2):288–94. 10.1097/01.mlg.0000250494.62826.6b 17277624

[17] You YS Chung CH Chang YJ Kim KH Jung SW Rho YS. Analysis of 120 Pectoralis Major flaps for head and neck reconstruction. Arch Plast Surg. (2012) 39(5):522–7. 10.5999/aps.2012.39.5.522 23094249 PMC3474410

[18] Lin YN Ou-Yang F Hsieh MC Lee SS Huang SH Chuang CH Use of extended pedicled transverse Rectus abdominis myocutaneous flap for extensive chest wall defect reconstruction after mastectomy for locally advanced breast cancer. Ann Plast Surg. (2020) 84(1S Suppl 1):S34–9. 10.1097/SAP.0000000000002188 31800552

[19] Küntscher MV Mansouri S Noack N Hartmann B. Versatility of vertical Rectus abdominis musculocutaneous flaps. Microsurgery. (2006) 26(5):363–9. 10.1002/micr.20253 16761268

[20] Longo B D'Orsi G Pistoia A Gagliano E Vannucchi L Natali GL T-inverted shaped Rectus abdominis myocutaneous (ti-Ram) flap for chest wall reconstruction. Plast Reconstr Regen Surg. (2022):64–8. 10.57604/PRRS-028

[21] Hanna WC Ferri LE McKendy KM Turcotte R Sirois C Mulder DS. Reconstruction after Major chest wall resection: can rigid fixation be avoided? Surgery. (2011) 150(4):590–7. 10.1016/j.surg.2011.07.055 22000169

[22] Leuzzi G Nachira D Cesario A Novellis P Petracca Ciavarella L Lococo F Chest wall tumors and prosthetic reconstruction: a comparative analysis on functional outcome. Thorac Cancer. (2015) 6(3):247–54. 10.1111/1759-7714.12172 26273369 PMC4448378

[23] Geissen NM Medairos R Davila E Basu S Warren WH Chmielewski GW Number of ribs resected is associated with respiratory complications following lobectomy with en bloc chest wall resection. Lung. (2016) 194(4):619–24. 10.1007/s00408-016-9882-3 27107874

[24] Talmi YP Benzaray S Peleg M Eyal A Bedrin L Shoshani Y Pulmonary function after Pectoralis Major myocutaneous flap harvest. Laryngoscope. (2002) 112(3):467–71. 10.1097/00005537-200203000-00012 12148856

[25] Tacconi F Ambrogi V Mineo D Mineo TC. The impact on quality of life after en-bloc resection for non-small-cell lung cancer involving the chest wall. Thorac Cancer. (2012) 3(4):326–33. 10.1111/j.1759-7714.2012.00132.x 28920274

[26] Maranetra KN Chuaychoo B Dejsomritrutai W Chierakul N Nana A Lertakyamanee J The prevalence and incidence of copd among urban older persons of Bangkok metropolis. J Med Assoc Thai. (2002) 85(11):1147–55. 12546310

[27] Sorotos M Firmani G Schiavone L Ricci A Santanelli di Pompeo F. Effects of diep flap-based breast reconstruction on respiratory function. J Plast Reconstr Aesthet Surg. (2023) 81:99–104. 10.1016/j.bjps.2023.02.025 37130446

[28] Katsura M Kuriyama A Takeshima T Fukuhara S Furukawa TA. Preoperative inspiratory muscle training for postoperative pulmonary complications in adults undergoing cardiac and Major abdominal surgery. Cochrane Database Syst Rev. (2015) 2015(10):Cd010356. 10.1002/14651858.CD010356.pub2 26436600 PMC9251477

